# Comparison of lipopolysaccharides composition of two different strains of *Helicobacter pylori*

**DOI:** 10.1186/s12866-017-1135-y

**Published:** 2017-12-04

**Authors:** Kristy Leker, Ivonne Lozano-Pope, Keya Bandyopadhyay, Biswa P. Choudhury, Marygorret Obonyo

**Affiliations:** 1 Division of Infectious Diseases, Department of Medicine, University of California San Diego, 9500 Gilman Drive, La Jolla, San Diego, California 92093 USA; 2 Glycotechnology Core Resources, University of California San Diego, 9500 Gilman Drive, La Jolla, San Diego, California 92093 USA

**Keywords:** *Helicobacter pylori*, Lipopolysaccharide, Chemical composition

## Abstract

**Background:**

*Helicobacter pylori* (*H. pylori)* is a Gram-negative, microaerophilic bacterium that is recognized as a major cause of chronic gastritis, peptic ulcers, and gastric cancer. Comparable to other Gram-negative bacteria, lipopolysaccharides (LPS) are an important cellular component of the outer membrane of *H. pylori*. The LPS of this organism plays a key role in its colonization and persistence in the stomach. In addition, *H. pylori* LPS modulates pathogen-induced host inflammatory responses resulting in chronic inflammation within the gastrointestinal tract. Very little is known about the comparative LPS compositions of different strains of *H. pylori* with varied degree of virulence in human. Therefore, LPS was analyzed from two strains of *H. pylori* with differing potency in inducing inflammatory responses (SS1 and G27). LPS were extracted from aqueous and phenol layer of hot-phenol water extraction method and subjected for composition analysis by gas chromatography – mass spectrometry (GC-MS) to sugar and fatty acid compositions.

**Results:**

The major difference between the two strains of *H. pylori* is the presence of Rhamnose, Fucose and GalNAc in the SS1 strain, which was either not found or with low abundance in the G27 strain. On the other hand, high amount of Mannose was present in G27 in comparison to SS1. Fatty acid composition of lipid-A portion also showed considerable amount of differences between the two strains, phenol layer of SS1 had enhanced amount of 3 hydroxy decanoic acid (3-OH-C10:0) and 3-hydroxy dodecanoic acid (3-OH-C12:0) which were not present in G27, whereas myristic acid (C14:0) was present in G27 in relatively high amount.

**Conclusion:**

The composition analysis of *H. pylori* LPS, revealed differences in sugars and fatty acids composition between a mouse adapted strain SS1 and G27. This knowledge provides a novel way to dissect out their importance in host-pathogen interaction in further studies.

**Electronic supplementary material:**

The online version of this article (10.1186/s12866-017-1135-y) contains supplementary material, which is available to authorized users.

## Background

*Helicobacter pylori (H. pylori)* is the most common bacterial pathogen in the world, infecting approximately 50% of the world’s population [1]. It colonizes the human stomach where it can persist for many years and if left untreated resulting in the development of peptic ulcer disease and gastric cancer [1–5]. Like other Gram-negative bacteria, the cell wall of *H. pylori* is composed of lipopolysaccharide (LPS). *H. pylori* LPS is suggested to contribute to its pathogenicity by contributing to colonization and persistence in the stomach [6–8]. LPS is an important cellular component of the outer membrane of Gram-negative bacteria, and has three distinct components: the core oligosaccharide, the lipid-A region, and the O-antigen [9]. The core domain contains an oligosaccharide such as heptose and attaches directly to the lipid-A region [10]. The lipid-A region generally consists of a phosphorylated glucosamine disaccharide surrounded by multiple hydrophobic fatty acid chains that help anchor the LPS into the bacterium while allowing the rest to protrude from the cell surface [11]. The O-antigen is a glycan polymer and is the outer most section of the LPS structure [11]. LPS can exist with or without its O-antigen side chains, making it smooth or rough LPS, respectively [12]. Each component of the LPS layer has its own unique function contributing to the bacteria’s survival in the host. The LPS is known to play an essential role in inducing the host’s activation of cytokine release and increased inflammatory response [9] thereby inducing a strong immune response in the host. The O-antigen of *H. pylori* strains has been reported to contribute to the virulence of this pathogen [13]. *H. pylori* is able to imitate carbohydrate structures present within the (host) human blood cells, secretions and particularly epithelial cells by incorporation of Lewis antigens into their O-chains [6]. In fact, Lewis antigens are expressed in over 80% of *H. pylori* strains tested [14].

There is a vast amount of structural diversity among Gram-negative lipid-A species. This is due in large part to the enzyme that modify the lipid A structure [15]. Previous reports have shown low immunological response to *H. pylori* LPS when compared to other Gram-negative bacteria such as *Escherichia coli (E. coli)* [16] and *Salmonella typhimurium* [17]. This low level immunologic response to LPS may explain why *H. pylori* infection is chronic lasting for decades [18]. There are also unusually high levels of genetic variation among the differing strains of *H. pylori* giving a varying degree of associated pathologies [19]. Associated virulence factors among the differing strains may also be attributed to the geographic location to where the strain was first isolated [20]. *H. pylori* strain G27, originally isolated from a patient at the Grosseto Hospital in Tuscany, Italy [21], contains a cytotoxin- associated pathogenicity island (cag-PAI) as well as the secreted vacuolating cytotoxin A (VacA) both of which correlate with the more severe associated pathologies such as gastric cancer [19]. The pathogenicity island is considered to be one of the major determinants of virulence in the *H. pylori* strains [22]. In addition, G27 is known to efficiently express Cag A [19, 21, 23, 24]**,** which is a virulence factor associated with increased risk of gastric cancer development [25, 26]. *H. pylori* Sydney Strain 1 (SS1), originally isolated from patients with peptic ulcers in Sydney, Australia [27] is a mouse-adapted strain that has a non-functional type IV secretion system (T4SS) [28] resulting in inability to secrete Cag A in epithelial host cells [29]. Epidemiologic studies have long reported strong association between secretion of CagA and the severity of *H. pylori* disease [20, 30]. In the present study, we compared LPS chemical composition of two strains of *H. pylori* with varying potency SS1 and G27. By comparing the chemical structures of LPS from SS1, a less potent strain with that of G27, a more potent strain, we can examine if differences in LPS composition contribute to differences in virulence of these strains. Whilst most of *H. pylori* LPS composition data [31–34] are chiefly based on the analysis of the aqueous layer of the phenol-water extraction procedure, we used both the aqueous and phenol phases of LPS extraction to achieve a complete composition of *H. pylori* LPS of the two *H. pylori* strains used in this study.

## Methods

### *H. pylori* strains and culture

*H. pylori* strains Sydney strain 1 (SS1) [27] and G27 (a kind gift from Dr. Guillemin, University of Oregon) were used in this study. *H. pylori* were routinely maintained on solid medium, Columbia agar (Becton Dickson, MD) supplemented with 5% laked blood (Hardy Diagnostics, CA) and Amphotericin B (2 μg/ ml) (Mediatech, VA) and grown at 37 °C under microaerophilic conditions (5% O_2_, 10% CO_2_, 85% N_2_) [35, 36]. For LPS extraction, *H. pylori* were subcultured into a liquid medium consisting of brain heart infusion broth (BHI, Becton Dickson, MD) supplemented with 5% fetal bovine serum (FBS) and cultured for twenty-four hours on a reciprocal shaker at 37 °C under microaerophilic conditions. Bacteria used for LPS extraction were in logarithmic phase of growth.

### LPS extraction

*H. pylori* strains grown in liquid culture were centrifuged (23,426 x g) for 30 min and the bacterial pellet washed twice in phosphate buffered saline (PBS). LPS was extracted by the standard Westphal method, more commonly known as the hot phenol-water extraction methods as described previously [37] with minor modifications. Briefly, *H. pylori* pellet was suspended in 20 mL endotoxin free molecular grade water followed by preheating in a water bath (68 °C) for 10 min with continuous stirring. A preheated 90% aqueous phenol solution (68 °C) was added slowly to the bacterial suspension and stirred vigorously for 30 min at 68^°^C. The reaction mixture was immediately cooled down to below 10 °C. The extracted mixture containing LPS was centrifuged at 1464 x g at 10 °C for 45 min to separate LPS according to the ratio of polysaccharide to lipid content and from other cellular components. LPS was obtained from both aqueous (LPS-AqL) and phenol (LPS-PhL) phase separately. The crude LPS mixtures were treated with DNase and RNase (Benzonase Nuclease, EMD-Millipore) and proteinase- K (Sigma) followed by extensive dialysis to remove the buffers from LPS as described previously [12]. The LPS was further purified by ultracentrifugation at 120, 000 g for 4 h at 4 °C (Beckman Ultracentrifuge) and the pellet was suspended in molecular biology grade endotoxin free water, lyophilized, weighed carefully, and resuspended in endotoxin free water at known concentrations. The LPS-AqL and LPS-PhL were used for SDS-PAGE and chemical characterization by gas chromatography – mass spectrometry (GC-MS, Agilent Technologies).

### SDS polyacrylamide gel electrophoresis (SDS-PAGE) analysis of *H. pylori* LPS

Electrophoretic profiles of both fractions of LPS (LPS-AqL and LPS-PhL) from each strain were determined by running samples in SDS-PAGE. Commercially available LPS (*E. coli* 0111 B4, Sigma Aldrich, MO) was used as a standard and a protein marker (Precision Plus, Bio-rad, CA) was used for LPS size localization. The gel was silver stained (Silver Quest Silver Staining Kit, Invitrogen, CA) according to the manufacturer’s instructions.

### Monosaccharide composition analysis of LPS

Composition analysis of LPS-AqL and LPS-PhL were performed using GC-MS as their methyl glycoside tri-methyl silyl derivatives (TMS) as previously described [38]. Briefly, 25 μg of LPS was spiked with 1 μg of Myo-inositol as an internal standard and methanolyzed using 1 M methanolic-HCl at 80 °C for 16 h. The reaction mixtures were cooled at room temperature. A nitrogen flush was used to remove any excess acid from the reaction mixtures. Methyl glycosides of amino-sugars were re-N-acetylated using a volume ratio of 4:1:1 of methanol: pyridine: acetic anhydride at 100 °C for 1 h. Finally, TMS derivatization was done using Tri-Sil reagent (Thermo Scientific) at 80 °C for 30 min. Samples were dried by dry nitrogen flush and TMS derivative of monosaccharides were dissolved in hexane and analyzed by GC-MS using a Restek-5 ms (Restek) capillary column. Ultrapure helium was used as carrier gas at a linear flow of 1.25 mL/min with a temperature gradient of 120 °C–10 °C for 1 min, 140 °C–2 °C for 1 min, 220 °C for 2 min, 5 °C for 1 min, and 230 °C for 4 min. Injector and transfer line temperature were set at 220 °C and 280 °C, respectively. Constituent monosaccharides in LPS were identified and quantified by comparing the retention time and mass fragmentation pattern with available authentic standards.

### Fatty acid composition analysis of LPS

Fatty acid composition analysis from phenol and aqueous layers of LPS, from SS1 and G27 were performed using GC-MS as fatty acid methyl ester (FAME) derivative. Briefly, 50 μg of LPS was treated with 1 M methanolic HCl. (Supelco, PA) at 80 °C for 16 h to vaporize excess acid followed by extraction of fatty acids using chloroform. Hydroxy fatty acids were further treated with Tri-Sil HTP reagent (Thermo Scientific) at 80 °C for 30 min to convert the hydroxyl group into tri-methyl ethers. Finally, samples were dissolved in hexane and analyzed by GC-MS using a Restek-5 ms (Restek) capillary column. Helium was used as a carrier gas at 1.1971 mL/min and a temperature gradient of 100 °C–5 °C for 1 min, 120 °C for 1 min, 3 °C for 1 min, and −230 °C for 4 min. Fatty acids were identified by their characteristic electron impact (EI) fragmentation pattern and retention times. The relative area percentage of the fatty acids are reported.

## Results

### Electrophoretic profile of LPS extracted from *H. pylori* strains, SS1 and G27

**Fig. 1:**
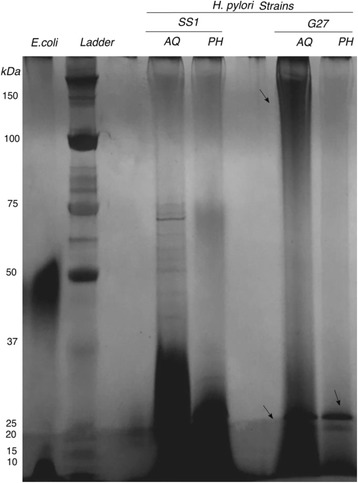
SDS-PAGE analysis of LPS aqueous and phenol fractions. LPS was extracted from both the aqueous (aq) and phenol (ph) fractions of *H. pylori* SS1 and G27 strains. *E. coli* strain 055:B5 was used as standard LPS. A protein ladder was used for band sizing. SDS samples were determined using 20ul of LPS extract and then visualized with periodic silver stain

LPS from *H. pylori* strains, SS1 and G27 was extracted from both aqueous and phenol phases. The silver-stained profiles of the LPS-AqL and LPS-PhL from SS1 and G27 were compared with *E. coli* as a control (Fig. 1). The LPS migration patterns between the two strains were distinct. In *H. pylori* SS1, although the aqueous and phenol layers were distinct from each other, they were within a similar range according to size. The majority of the LPS in each fraction stayed within 37 kDa or lower. In comparison, the G27 strains appeared to have significant differences not only to the SS1 strain but within their own aqueous and phenol layer of LPS. G27 aqueous layer LPS had two distinct LPS smears, one with molecular weight ranging from approximately 80- >100 kDa and another low molecular weight starting around 30 kDa and lower. G27 phenol layer LPS was also unique, since it featured one distinct band at 25 kDa and low molecular weight distribution near 5 kDa and lower. All of the strains showed a dramatic difference from the control strain *E. coli* that had its major LPS within the aqueous layer at 25 kDa range and another band at the 50 kDa range.

### GC-MS analysis of monosaccharides obtained from aqueous and phenol phase LPS

**Fig. 2:**
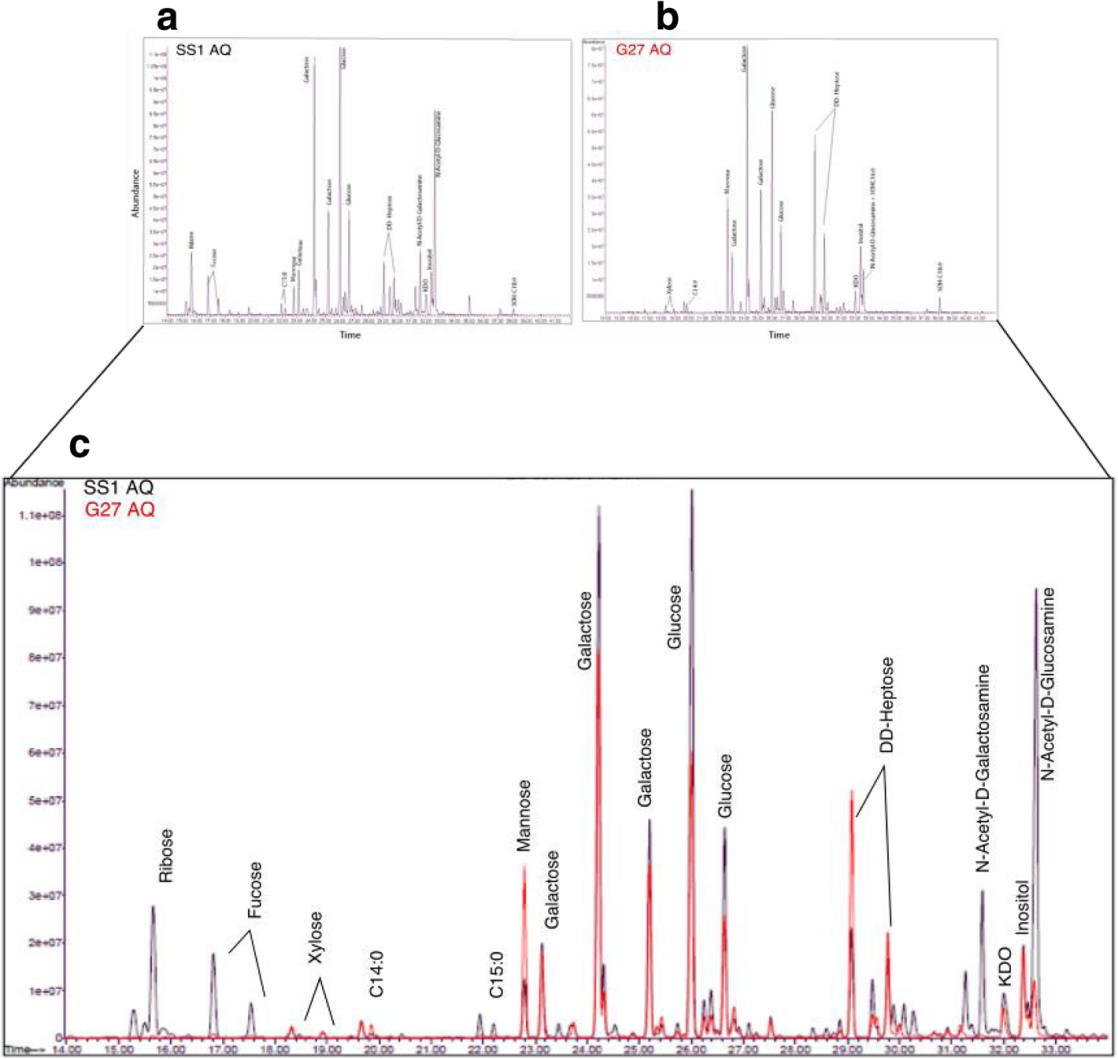
LPS composition analysis. LPS from aqueous fractions of *H. pylori* strains, G27 and SS1 were analyzed by GC-MS as both FAME and Tri-Sil FAME derivatives of hydroxy fatty acids from (**a**) SS1 (**b**) G27 and an overlay (**c**) depicting both SS1 (black) and G27 (red) is shown for comparison

**Fig. 3:**
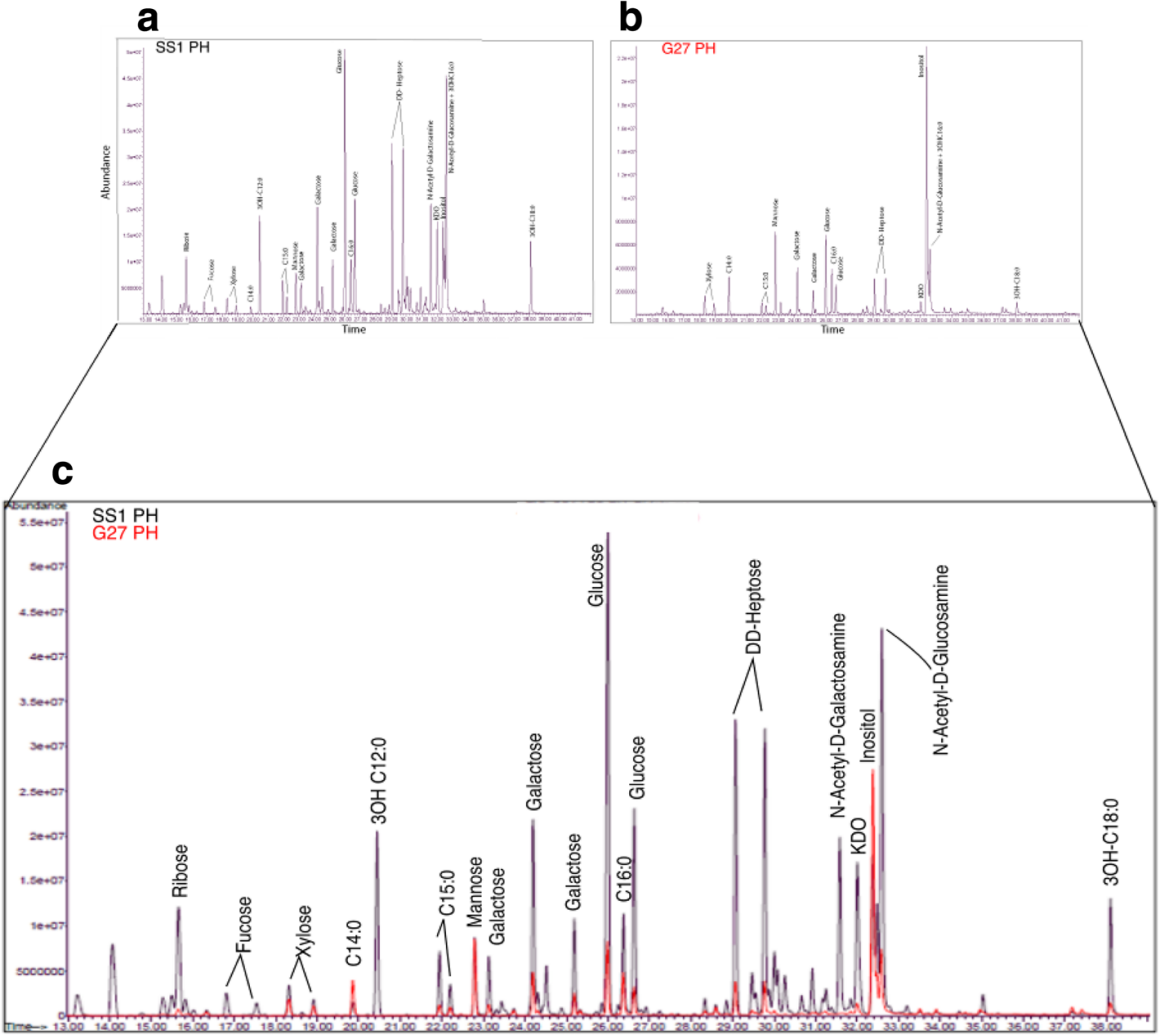
Composition analysis of LPS extracted from phenol fractions. *H. pylori* strains, SS1 (**a**) and G27 (**b**) were analyzed by GC-MS analysis as FAME and Tri-Sil FAME derivatives. An overlay (**c**) is shown with SS1 (black) and G27 (red) for comparison

The LPS carbohydrate composition of from both strains is summarized in Figs 2 and 3, Table 1, and Additional file 1: Figure S1. Monosaccharides that were present at significant levels in different LPS fractions from both strains included xylose (Xyl), mannose (Man), galactose (Gal), glucose (Glc), heptose (Hep), and N-Acetylglucosamine (GlcNAc); however, they were present at varying degrees depending on the strain and the layer. 2-keto-3-deoxyoctonate (KDO) the inner core sugar connecting Lipid-A portion to the inner core oligosaccharide of LPS is present in all fractions in different proportion. The other interesting difference is the presence of significant amount of GalNAc in both aqueous and phenol layer LPS of SS1, which is completely absent in G27. GC-MS analysis of *E. coli* LPS strain 0113 was performed as a standard reference (Additional file 1: Figure S2).

**Table 1:** Mole percentage of monosaccharides present in LPS

	Abbreviation	SS1 - Aqueous layer	SS1 - Phenol Layer	G27 - Aqueous Layer	G27- Phenol Layer
Ribose	Rib	14.63	18.71	–	9.58
Rhamnose	Rha	7.46	–	–	–
Fucose	Fuc	10.34	2.69	0.79	–
Xylose	Xyl	1.02	2.41	2.09	12.74
Mannose	Man	1.55	1.62	10.03	13.94
Galactose	Gal	22.84	6.26	34.73	13.35
Glucose	Glc	17.36	11.31	18.38	13.74
N-Acetyl-D-galactosamine	GalNac	18.16	16.53	–	–
N-Acetyl-D-glucosamine	GlcNac	2.71	14.08	11.64	20.1
Heptose	Hep	3.93	10.6	16.28	9.71
3-deoxy-α-D-mannooctulosonic acid	KDO	5.31	15.78	6.06	6.83
Amount (μg/ 100 ul)		254.69	179.71	119.78	13.24

LPS from Helicobacter pylori strains SS1 and G27 were purified into aqueous (Aq) and phenol (Ph) layers and were separately subjected to composition analysis. The sugar composition values of aqueous and phenol layers are presented as mole percentages of monosaccharides obtained in fractions of the total amount that is presented in μg/100ul

### Fatty acid composition analysis of LPS

The fatty acid composition of both strains’ LPS analyzed by GC-MS is summarized in Table 2. The two fatty acids that were present in both strains from both layers were palmitic acid (C16:0) and stearic acid (C18:0). However, there were significant differences between the two strains in regard to their other fatty acid composition. The aqueous and phenol layer LPS from G27 strain had a similar presence of fatty acids, except for pentadecylic acid (C15:0), which was absent in the G27 aqueous layer. Unlike the G27 strain, SS1 strain had significant differences in fatty acid composition between aqueous and phenol layers. The fatty acid distribution in SS1 aqueous layer LPS showed the presence of only a few fatty acids, namely pentadecylic acid (C15:0), palmitic acid (C16:0), and stearic acid (C18:0). On the other hand, the phenol layer LPS had a significant amount of 3-hydroxy fatty acids with lower chain length such as 3-hydroxy decanoic acid (3-OH C10:0) and 3-hydroxy dodecanoic acid (3-OH C12:0). The other noticeable difference between the strains was the presence of relatively high amount of myristic acid (C14:0) in G27 as compared to the SS1 strain. Hence combining the monosaccharide and fatty acid composition data along with LPS molecular heterogeneity as shown in SDS-PAGE it can be concluded that there is a significant structural difference in the LPS layer of both strains.

**Table 2:** Relative are percentage of fatty acids present in LPS

		SS1 - Aqueous layer	SS1 - Phenol Layer	G27 - Aqueous Layer	G27- Phenol Layer
3-Hydroxy Decanoic Acid	3OH-C10:0	–	10.86	–	–
3-Hydroxy Dodecanoic Acid	3OH-C12:0	–	17.11	–	–
3-Hydroxy Hexadecanoic Acid	3OH-C16:0	–	23.4	31.22	21.68
3-Hydroxy Octadecanoic Acid	3OH-C18:0	–	13.65	19.43	7.247
Lauric Acid	C12:0	–	3.87	–	–
Myristic Acid	C14:0	–	1.43	11.1	20.24
Pentadecylic Acid	C15:0	29.9	8.84	–	11.33
Palmitic Acid	C16:0	45.83	10.14	21.07	22.74
Stearic Acid	C18:0	24.26	10.7	17.19	16.75

LPS from *H. pylori* strains, SS1 and G27 were purified into aqueous (Aq) and phenol (Ph) layers and were separately subjected to composition analysis. The fatty acid composition for the aqueous and the phenol layers were performed using the area under the corresponding fatty acids and is a relative percentage calculated as a percentage of that particular fatty acid in the total mixture

## Discussion

LPS is a vital structural component of all Gram-negative bacteria and has significant effect on the endotoxin activity of the bacteria. Knowledge of LPS composition in addition to indicating the degree of LPS potency may also lead to the discovery of new treatment methods, thereby underscoring the importance of performing a complete LPS composition analysis. In the present study, we used both the aqueous and phenol phases of LPS hot phenol/water extraction to determine the composition of *H. pylori* LPS. This is in contrast to previous *H. pylori* LPS composition studies, which used only the aqueous phase to define the composition of LPS [31–34, 39, 40]. A drawback of using only one phase is the potential of missing some molecules. We compared the LPS composition of *H. pylori*, strains SS1 and G27. *H. pylori*, SS1 is a commonly used strain and the chemical structure of its LPS is known [41]. The LPS composition of G27 was unknown when we commenced this study but was published this year [40]. However, similar to previous studies on *H. pylori* LPS composition, this study also used only the aqueous phase for analysis.

We show that *H. pylori* LPS is unique and like Leptospira LPS separates into two phases [12]. We found that a high number of hydroxyl fatty acids from SS1 separated into the phenol phase of hot phenol-water extract compared to G27. This indicates that the distribution of *H. pylori* LPS in the phenol and aqueous layers may be dependent on the *H. pylori* strain. These results therefore underscore the importance of analyzing LPS isolated from both phases. The ratio of hydrophilic saccharide and hydrophobic lipid portions of LPS influence their distribution in the aqueous and phenol phases [42]. SDS-PAGE analysis of LPS showed clear differences in the LPS composition of the two *H. pylori* strains. The LPS composition of the G27 strain was generally similar in the aqueous and the phenol layer; whereas, for the SS1 strain, the aqueous layer had a larger amount of different sized molecules. The biggest difference in *H. pylori* composition between layers (aqueous and phenol) was in the SS1 strain with the detection of many low molecular weight fatty acids in the phenol layer and none in the aqueous layer. This may be associated with the thick smear we observed at the bottom of the phenol SS1 in the silver-stained gel but was absent in G27. Another noticeable difference was the presence of copious amounts of low molecular weight LPS molecules in the phenol phase of SS1 that was not visible in G27. This low molecular weight material possibly represents the highly defined LPS material such as the short-chained fatty acids that was detectable by GC-MS. We detected core constituents previously found in *H. pylori* SS1 LPS including ribose, fucose, glucose, galactose and DD- heptose [8]. Heptose was present in both strains at significant amounts, although the SS1 aqueous layer had significantly less heptose than the G27 aqueous layer. Heptose is a common monosaccharide within the LPS core and has been proven lethal when deleted in *H. pylori* [43] emphasizing the importance of heptose for LPS inner core for membrane integrity and viability. In *H. pylori* LPS, the heptose moiety is DD-heptose, which is found within the inner core of the LPS [7].

Other monosaccharides, such as glucose and galactose, which reside in the outer core, were present in both strains. However, in general the G27 strain had higher concentrations of monosaccharides that contribute to the core of the LPS molecule while *H. pylori* SS1 had increased concentrations of monosaccharides that are directly attributed to the O-antigen region of the LPS, such as fucose. Fucose was very prominent in SS1 at high concentrations in the aqueous phase and in small quantities in the phenol phase. In the G27 strain the amount of fucose detected was negligible in the aqueous phase and undetectable in the phenol phase. Usually found attached to the O-antigen structure, this sugar generates Lewis antigens, which are also expressed in the gastric epithelium [44]. Fucose is therefore commonly known for playing a role in the host immune response evasion [45]. This could contribute to the adaptability of the SS1 strain in colonizing mice very efficiently. Further studies are needed to test the role of fucose in colonization ability. A rather interesting sugar observed in large amounts in the SS1 strain that was not present in G27 was GalNAc, which plays a role in glycosylation of mucins in the human gastric mucosa [46]. Generally, in a healthy human gastric mucosa, mucins are produced that are either secreted or membrane-bound that help create a physical barrier of the gastrointestinal tract [47]. Largely, the mucins produced in the gastric mucosa include MUC1, MUC5AC and MUC6 [46]. These mucins have a tandem repeat in their amino acid sequence and these sequences are highly glycosylated with GalNAc O-linkages [47]. GalNAc is a derivative of galactose usually found in humans. This bacterial homologue of human blood group A transferase has been shown to accept blood group A antigens thereby showing an exceptional ability to synthesize human blood group A antigens and essentially mask itself from the human immune system [48]. GalNAc is necessary for intercellular communication and its presence only in SS1, which is a mouse adapted strain suggests the efficient adaptation of this strain to the mammalian immune system by mimicry as a means of concealment. The monosaccharide KDO is another provably essential monosaccharide for *H. pylori* LPS composition. Our analysis showed differences in the monosaccharide KDO between the *H. pylori* strains, SS1 and G27. KDO functions as a link between the essential heptose moieties of the inner core and the Lipid-A domain [49]. Whereas, the G27 strain showed similar concentrations of KDO in both the aqueous and the phenol layers, the SS1 strain did not have a significant amount of KDO in the aqueous layer. The phenol layer of SS1, however, had larger amounts of KDO than both layers of G27. Further studies are needed to understand the possible importance of the large amounts of KDO we observed in the SS1 strain compared to the G27 strain. In the present study, while our LPS analysis showed presence of mannose in the G27 strain, in contrast, it was not detected in a recent study by Li et al. [40]. Mannose has been detected in other *H. pylori* strains including NCTC 11637, S-24, and C-5437 [50]. The reasons for this contradictory finding are currently unknown but it is possible the different growth conditions of *H. pylori* were a factor. We cultured *H. pylori* in liquid media (BHI) prior to LPS extraction. In contrast, Li et al. [40] cultured *H. pylori* on solid media, Columbia Blood Agar. Indeed, *H. pylori* growth medium composition has been shown to impact the expression of *H. pylori* antigens, especially LPS [51].

Fatty acids contribute to the Lipid-A region of the LPS structure. For most organisms, the fatty acid lengths are C10 - C14 with a few having higher fatty acid lengths of around C16 - C18 [52]. The fatty acid moiety lengths for *H. pylori* are generally around C16-C18 [53]. Hydroxy fatty acids are components of the Lipid-A region of the LPS structure so they are generally good indicators of LPS integrity [54]. A major disparity in LPS composition between *H. pylori* strains, SS1 and G27 was the distribution of lower molecular weight fatty acids including lauric acid (C12:0), β-hydroxy lauric acid (3OH:C12:0), and β-hydroxy decanoic acid (3OH:C10:0), which were observed in the SS1 strain and were not detected in the G27 strain. In addition, the GC-MS analysis of G27 showed more high weight molecular weight fatty acids with little or no low molecular weight fatty acids. This may signify the presence of heavy oligosaccharides, which are usually present in bacteria that produce the smooth form of LPS. In contrast, the SS1 strain LPS was composed of more low molecular weight fatty acids, which suggest a closer association to a rough form of LPS. Previous investigations of *H. pylori* strains indicated that many long-chain fatty acids (higher molecular weight) yield smooth LPS while those with shorter chain fatty acids (lower molecular weights) yield rough LPS [50, 55]. High molecular weight, or smooth LPS, generally have a repeating O-chain which is high in molecular weight [56]. In contrast, rough LPS lacks an O-chain and instead has a short-chain oligosaccharide in place of the O-chain giving it a shortened, rough appearance. The different forms of *H. pylori* LPS have been associated with the ability of these bacterial organisms to display antigenic diversity, which allow them to evade the host immune systems.

## Conclusions

The purpose of this experiment was to analyze the differences in the composition of LPS between two *H. pylori* strains of varying potency through a detailed biochemical composition analysis. We compared LPS composition of SS1 to G27, which had not been analyzed before we commenced our study. *H. pylori* SS1 LPS had more sugars and fatty acids compared to the G27 LPS. The significance of these differences in LPS composition of these two *H. pylori* strains is unknown at this time. The greatest differences between the strains SS1 and G27 were seen in the foundational structures of the strains, such as the Lipid-A domain and the amount of O-antigen side chains that were present. Although further investigation is needed to confirm the importance of certain sugars and fatty acids on the potency of the strain, the differences between the two strains are clearly evident. Future studies will define Lipid-A and O-antigenic structure of LPS to improve the comprehension of the mechanisms of pathogenesis.

## Additional file

Additional file 1: Figure S1. An inverted overlay of LPS composition analysis from *H. pylori* strains, SS1 and G27. The aqueous (a) and phenol (b) fractions depicts the differences in peak heights of SS1 (black) and G27 (red) using GC-MS analysis of FAME and Tri-Sil FAME derivatives. **Figure S2**. GC-MS analysis of *E. coli* strain 0113. *E. coli* LPS was analyzed as standard reference. (PDF 530 kb)

## Abbreviations

3OH:C10:0: 3-hydroxydecanoic acid
3OH:C12:0: 3-hydroxydodecanoic acid
3OH:C18:0: 3-hydroxy octadecanoic acid
AA: Alditol acetate derivative
AQ: Aqueous
BHI: Brain-heart infusion broth
C12:0: Lauric acid
C14:0: Myristic acid
C15:0: Pentadecyclic acid
C16:0: Palmitic acid
C18:0: Stearic acid
Cag-PAI: Cytotoxin associated pathogenicity island
DMB: 4,5 methylenedioxy-1, 2-phenylenediamine dihydrochloride
*E. coli*: *Escherichia coli*
EI: Electron impact
FAME: Fatty acid methyl ester
FBS: Fetal bovine serum
Gal: Galactose
GalNAc: N-Acetyl Galactosamine
GC-MS: Gas chromatography-mass spectrometry
Glc: Galactose
GlcNAc: N-Acetyl Glucosamine
H. pylori: Helicobacter pylori
Hep: Heptose
INO: Myo-inositol
KDO: 2-keto-3-deoxyoctonate
LPS: Lipopolysaccharide
Man: Mannose
PH: Phenol
Rha: Rhamnose
SS1: Sydney strain 1
TFA: Trifluoroacetic acid
TMS: Tri-methyl sil
Tri-Sil: Trimethyl silyl ether
VacA: Vacuolating cytotoxin A
Xyl: Xylose
