# Supplementary material for: Comparison of lipopolysaccharides composition of two different strains of Helicobacter pylori
Source: BMC Microbiol. 2017 Dec 4;17:226. doi: 10.1186/s12866-017-1135-y (PMC5715995; doi:10.1186/s12866-017-1135-y)
Supplement: Additional file 1: Figure S1. — An inverted overlay of LPS composition analysis from H. pylori strains, SS1 and G27. The aqueous (a) and phenol (b) fractions depicts the differences in peak heights of SS1 (black) and G27 (red) using GC-MS analysis of FAME and Tri-Sil FAME derivatives. Figure S2. GC-MS analysis of E. coli strain 0113. E. coli LPS was analyzed as standard reference. (PDF 530 kb) [file 12866_2017_1135_MOESM1_ESM.pdf]

Supplementary Data

Figure Legends

Figure S1. An inverted overlay of LPS composition analysis from *H. pylori* strains, SS1 and G27. The aqueous (a) and phenol (b) fractions depicts the differences in peak heights of SS1 (black) and G27 (red) using GC-MS analysis of FAME and Tri-Sil FAME derivatives.

Figure S2. GC-MS analysis of *E. coli* strain 0113. *E. coli* LPS was analyzed as standard reference.

Figure S.1

A

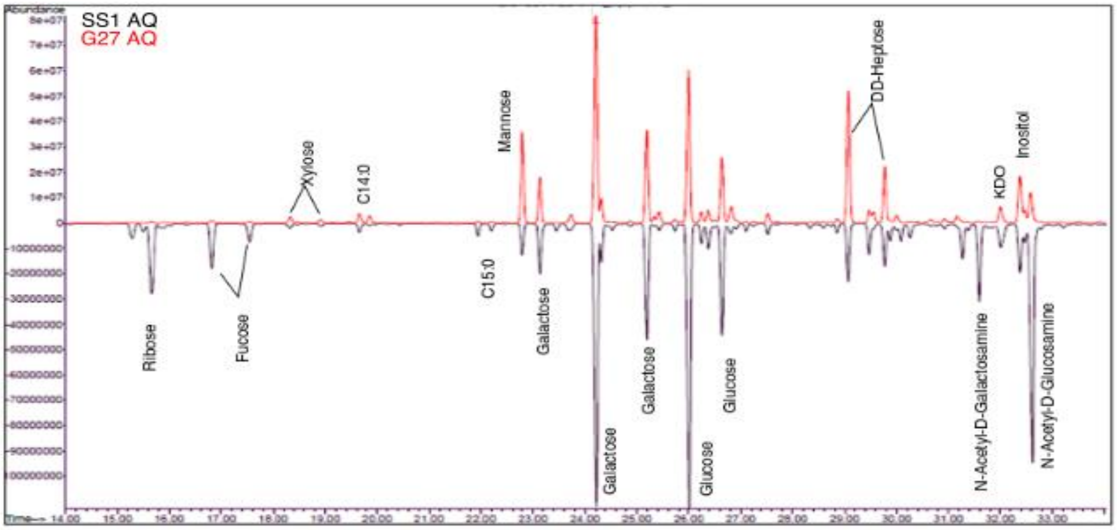

B

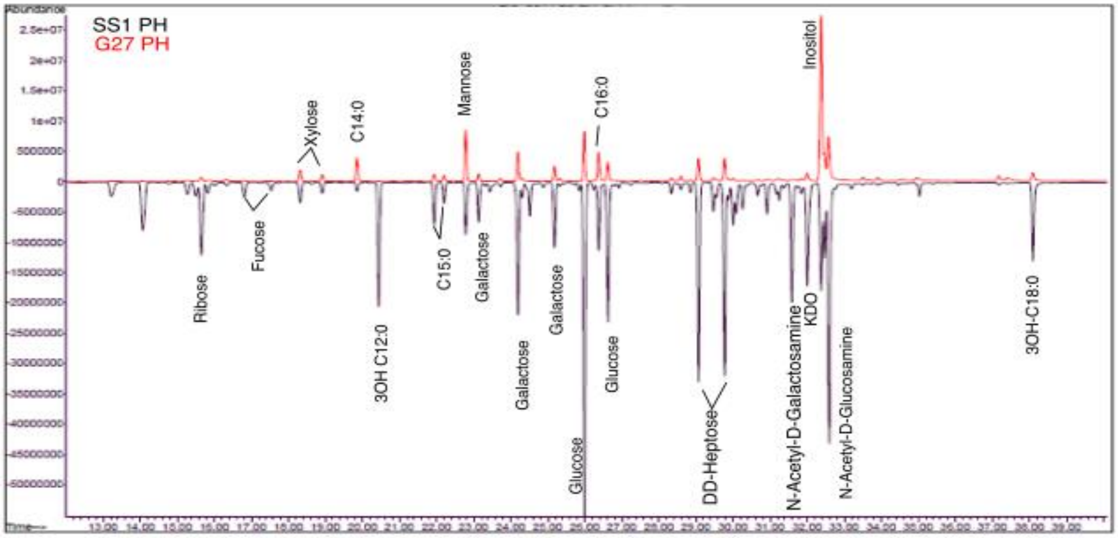

Figure S2.

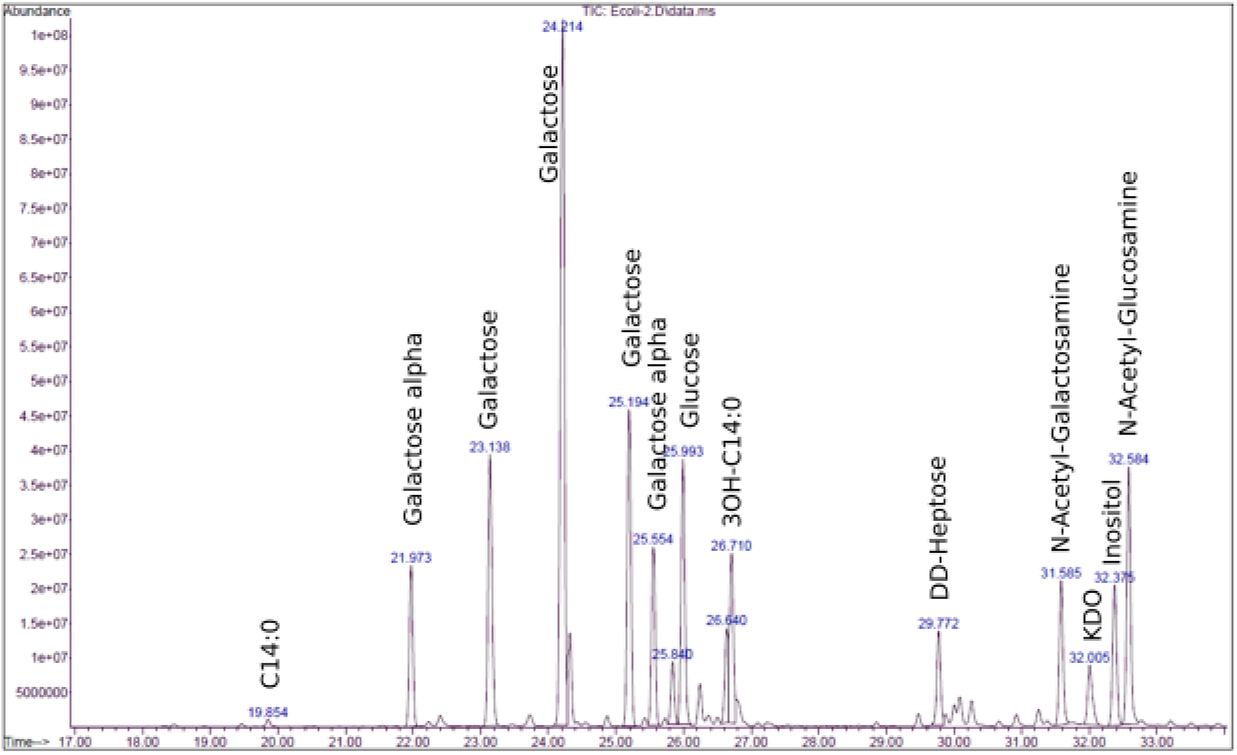

Abundance
TIC: Ecoli-2.D\data.ms
1e+08
9.5e+07
9e+07
8.5e+07
8e+07
7.5e+07
7e+07
6.5e+07
6e+07
5.5e+07
5e+07
4.5e+07
4e+07
3.5e+07
3e+07
2.5e+07
2e+07
1.5e+07
1e+07
5000000
Time--> 17.00 18.00 19.00 20.00 21.00 22.00 23.00 24.00 25.00 26.00 27.00 28.00 29.00 30.00 31.00 32.00 33.00
C14:0
19.854
Galactose alpha
21.973
Galactose
23.138
Galactose
24.214
Galactose
25.194
Galactose alpha
25.554
25.840
Glucose
25.993
3OH-C14:0
26.640
26.710
DD-Heptose
29.772
N-Acetyl-Galactosamine
31.585
KDO
32.005
Inositol
32.375
N-Acetyl-Glucosamine
32.584
